# Address by P. G. C. Hunt, at the Opening of the Indiana State Dental Association

**Published:** 1873-04

**Authors:** 


### Address by P. G. C. Hunt, at the Opening of the Indiana State Dental Association.

*Gentlemen of the Indiana State Dental Association*:—In glancing at the changes in dentistry, made during the past five years, it is certainly gratifying to see the advancement that has been made. This is now noticeable perhaps more in the operative department. Here we see a strong determination to elevate the standard of dental operations; so rapid have improvements followed one another, that we look back, and wonder how we used to succeed in any but the most simple cases. If I were now compelled to make a retrograde movement, to be deprived of the use of heavy foils, the mallet, and coffer-dam rubber, I should certainly seek some other calling.

Your Executive Committee have arranged for you a most excellent list of subjects for discussion; and I will not long detain you from their consideration. I wish to call your attention to the action of the other state societies, who have taken steps to memorialize Congress for the appointment of dentists in the army and navy. I should like to see you also act in this matter.

I would also recommend that in your wisdom, you devise some means by which there might be incorporated in our public school books, more thorough teachings in regard to the anatomy and physiology of the teeth, their importance, and the proper treatment to insure their preservation, etc.

I would further recommend the continuance of the old, or the appointment of a new committee on the subject of a state law to regulate the practice of dentistry. I am of the opinion that a law of this kind would contribute greatly to the well-being of the people of our State; and when understood, would meet with their hearty approval. There are certainly not the difficulties in the way of legislation as regards dentistry that there is in medicine, for there they have different schools claiming attention. Not so in dentistry; here it is simply a question of professional knowledge or ignorance.

Gentlemen, I return you thanks for your kind attention. Be courteous in debate and generous to each other. In our discussions let us search for and hold fast to truths, and leave all personal strife out of our meetings.

